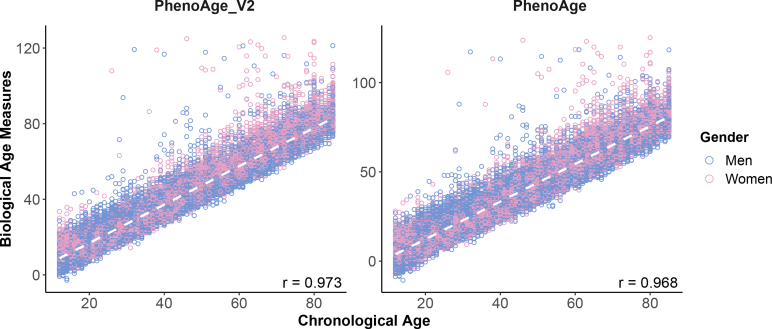# Phenotypic age mediates the associations between platelet-to-lymphocyte ratio and all-cause and cause-specific mortality: A prospective cohort study

**DOI:** 10.1016/j.heliyon.2024.e41506

**Published:** 2024-12-27

**Authors:** Xiangjun Li, Jia Wang, Mengqi Zhang, Yujing Li, Xiaoxuan Li, Jiaqi Zhang, Lihua Zhang, Yixuan Zhang, Zhenkang Qiu

**Affiliations:** a Breast Center, The Affiliated Hospital of Qingdao University, Qingdao, Shandong, China; b Department of Gastroenterology, The Affiliated Hospital of Qingdao University, Qingdao, Shandong, China; c Department of Oncology, Key Laboratory of Cancer Molecular and Translational, The Affiliated Hospital of Qingdao University, Qingdao, Shandong, China; d Interventional Medical Center, The Affiliated Hospital of Qingdao University, Qingdao, Shandong, China; e Department of Pathology, The First Hospital of China Medical University, College of Basic Medical Sciences of China Medical University, Shenyang, Liaoning, China; f Department of Medicine, Qingdao University, Qingdao, Liaoning, China

**Keywords:** NHANES, Platelet-to-lymphocyte ratio, Phenotypic age, All-cause mortality, Cause-specific mortality

## Abstract

**Objectives:**

The platelet-to-lymphocyte ratio (PLR) is a novel indicator of inflammation, but research on the links and mechanisms between the PLR and long-term health conditions is lacking. This study aimed to evaluate the relationship between phenotypic age (PhenoAge) mediated PLR and mortality among US adults.

**Methods:**

A total of 37,182 participants from the National Health and Nutrition Examination Survey (NHANES) database (1999–2018) were included to evaluate the PLR's relevance to survival by Cox regression models. The associations between the PLR and mortality were apparent using restricted cubic spline regression. Mediation analyses were conducted to investigate the mediated effects of PhenoAge on the associations of PLR with mortality.

**Results:**

Compared to the PLR in Quintile 1 participants, the multivariable-adjusted Cox model showed the PLR in Quintile 5 was linked with greater risks of death from all-cause (*HR*, 1.14; *95 % CI*: 1.04–1.25), cardiovascular disease (CVD) (*HR*, 1.26; *95 % CI*: 1.01–1.57) and respiratory disease (*HR*, 1.98; *95 % CI*: 1.35–2.90). The risk of death from cancer was approximately 28 % lower for participants with the PLR in the fourth quintile. Restricted cubic splines showed the U-shaped relationships between PLR and all-cause and cancer mortality, and the positively linear relationships between PLR and cardiovascular disease (CVD) and respiratory mortality. Moreover, mediation analysis revealed that PhenoAge partially mediated 45.33 %, 44.26 %, and 15.35 % of the associations of PLR with all-cause, CVD, and respiratory disease mortality, respectively.

**Conclusion:**

The PLR, a valuable index that should be recommended for use, was independently linked with all-cause and cause-specific mortality, with PhenoAge playing a partial mediating role in the relationships.

## 1 Background

As a novel indicator of systemic inflammation, the platelet-to-lymphocyte ratio (PLR) measures the ratio of platelets to lymphocytes in peripheral blood [1,2]. The PLR provides more details regarding thrombosis and inflammation pathways than lymphocyte or platelet counts alone, and it has a higher capacity to predict coronary atherosclerotic load [3,4]. The PLR is an indicator of systemic inflammation, an accurate prognostic predictor, and a reliable indicator of the therapeutic response in patients with various malignancies [[5], [6], [7], [8]]. PLR levels have been linked to the diagnosis and prognosis of infectious diseases such as bacteremia, sepsis, and community-acquired pneumonia in previous investigations [[9], [10], [11]]. Elevated PLRs have also been observed in individuals with chronic conditions such as autoimmune disorders, prediabetes, type 2 diabetes, and metabolic syndrome [12,13]. These results imply that PLR is a risk factor for various disorders.

It has been shown that Phenotypic Age (PhenoAge) [14], a unique multi-system-based aging measure, accurately captured mortality and morbidity risk across the entire US population and several subpopulations, even in healthy individuals. PhenoAge is an advanced biomarker that captures multiple physiological systems' deterioration, including inflammation markers. It reflects "biological age," integrating factors like white blood cell count, albumin, and other markers of physiological decline. PhenoAge is intended to identify persons at risk for a range of chronic illnesses or degenerative conditions by capturing age-related dysregulation. It can also be utilized to shed light on the genetic and environmental factors that influence how quickly people age in fundamental and observational research. Zuyun Liu et al.’s research indicated that PhenoAge was significantly associated with all-cause mortality after adjusting for chronological age and sex [15].

However, only a few clinical investigations have explored the relationship between PLR levels and long-term health consequences. Whether the PLR relates to a higher mortality risk in the normal population remains unknown. This research examined the relationship between PhenoAge mediated PLR levels and long-term all-cause and cause-specific mortality among US individuals using an extensive nationally representative dataset from the National Health and Nutrition Examination Survey (NHANES) [16].

## 2 Methods

### 2.1 Study population and design

The NHANES is a nationally representative study of noninstitutionalized US demographic groups with a stratified, multistage design undertaken by the Centers for Disease Control and Prevention (CDC) and the National Center for Health Statistics (NCHS [17]). The NHANES survey employs a complicated, multistage, nationwide probability sample survey methodology each year. All participants provided their consent in writing after being fully informed.

**Fig. 1:**
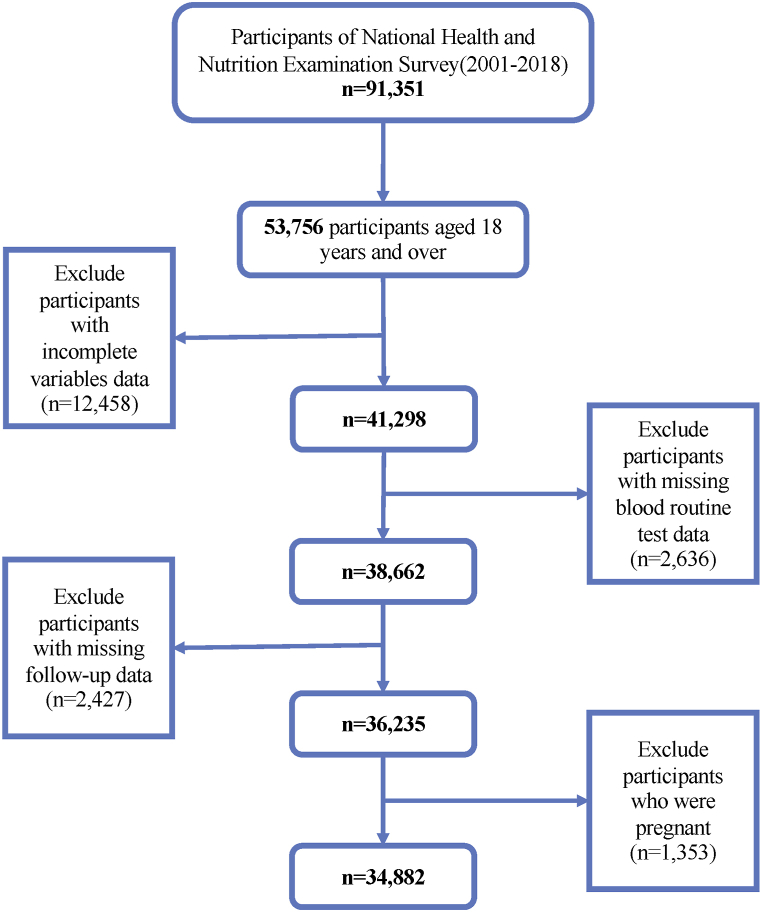
The research flowchart. Abbreviations: PLR, Platelet-lymphocyte ratio.

As cross-sectional data, 10 NHANES cycles (1999–2018) were used in this study. The National Death Index [18] (NDI) mortality data were combined to construct this cohort study. Initially, there were 55,081 participants under 20 years of age being eliminated. A total of 1541 pregnant women were eliminated. Furthermore, 2721 participants were excluded due to a lack of follow-up data. In addition, 2603 participants were disqualified because routine blood test data were not discovered or missing. 5, 082 participants with incomplete PhenoAge data were excluded. Following this, 5952 participants who did not complete the variable data were excluded. Ultimately, 37,182 participants were included in the analyses. The sample screening procedure is depicted in Fig. 1.

### 2.2 Assessment of outcomes

The NCHS provided Public-Use Linked Mortality Files. The mortality situation was determined by connecting the unique study identifier to the NDI (last follow-up on December 31, 2019, updated in 2022) [19]. According to the International Statistical Classification of Diseases codes, the causes of death were identified (tenth revision). The International Classification of Diseases 10th Revision (ICD-10) [20] was used to classify participants using the information on the major cause of death. The main findings for this study were mortality from all causes, CVD (cardiovascular diseases) (codes I00-I09, I11, I13, I20-I25, I26-I51, and I60-I69), cancer (codes C00-C97), and respiratory disease (codes J40-J47 and J09-J18).

### 2.3 Assessment of the PLR

Following established methodology and methods, platelet and lymphocyte counts were acquired from peripheral whole blood tests. The Laboratory Procedure Manual for the NHANES fully describes the test's operation method [21]. The reported levels for platelets and lymphocytes were 1000 cells/μL. By dividing the platelet count by the lymphocyte count, the PLR was computed.

### 2.4 Ascertainment of PhenoAge

PhenoAge [14] is computed from an algorithm derived from a multivariate analysis of mortality hazards. A “mortality score” was computed using a selected biomarker set and age, and then converted to a biological age value. Other definitions and measurements used in this study are provided in the Supplementary Methods.

### 2.5 Ascertainment of covariates

Detailed information on covariates is shown in the Supplementary Methods.

### 2.6 Statistical analyses

Following the NHANES analytical and reporting requirements, all statistical analyses considered complicated survey design characteristics, incorporating stratification, clustering, and sample weights. Person-years were calculated from the enrolment date to the date of death or censoring, whichever occurred first. HRs and 95 % CIs were used to display statistical indicators. We built three weighted Cox regression models to investigate the connection between the PLR and mortality. The potential mediating effects of PhenoAge on the associations of LE8 scores and mortality were estimated by a regression-based causal mediation analysis using the R package “Regmedint” [22]. The direct effect represented the effects of PhenoAge on mortality without a mediator. The indirect impact described the effects of PhenoAge on mortality through the mediator. The mediating proportion was calculated using indirect effect divided by total impact. A complete mediation effect is defined as when the indirect effect is statistically significant and the direct effect is not. Partial mediation effect is defined as when both indirect and direct effects are statistically significant. Unmediated effect is defined as when the indirect effect is not statistically significant and the direct effect is statistically significant. Other statistical analysis methods are described in the Supplementary Methods.

This study used *P* < 0.05 to evaluate statistical significance for all two-sided statistical tests. Analyses were performed using R 4.2.2 software.

## 3 Results

### 3.1 Population characteristics

According to the NHANES database, 37,182 adult participants were enrolled in this study (1999–2018). Participants were separated into five groups according to the PLR: Quintile 1 (≤89.62), Quintile 2 (89.63–110.39), Quintile 3 (110.40–131.25), Quintile 4 (130.26–162.07), and Quintile 5 (≥162.08). The average age of all participants was 47.70 ± 0.20 years (mean age and standard error), with females (50.57 %) slightly outnumbering males (49.43 %). Participants in Quintile 5 were more likely to be older, males, non-Hispanic white, non-smokers, non-alcohol users, obese, hypertension patients, cancer patients, and CVD patients, and have a higher education level, income, and diet quality (all *P* < 0.001). The baseline features of the research population based on the PLR are shown in Table 1.

**Table 1:** Demographic and clinic characteristics according to PLR levels. NHANES 1999–2018.^a^.

Characteristics	Total Adults	PLR					P value
	(N = 37,182)	Quintile 1 <89.62 (N = 7430)	Quintile 2 89.63–110.39 (N = 7445)	Quintile 3 110.40–131.25 (N = 7441)	Quintile 4 130.26–162.07 (N = 7429)	Quintile 5 ≥162.08 (N = 7437)	
**Age, y, mean (SE)**	46.70(0.20)	45.84(0.31)	45.36(0.31)	46.00(0.34)	46.67(0.33)	49.47(0.33)	<0.001
**Female, n (%)**	18443(50.57)	3238(44.55)	3560(48.88)	3679(49.66)	3877(53.38)	4089(55.59)	<0.001
**Race/ethnicity, n (%)**							<0.001
Non-Hispanic White	17631(71.39)	3104(65.35)	3320(69.47)	3541(72.65)	3720(72.68)	3946(76.05)	
Other	19551(28.61)	4326(34.65)	4125(30.53)	3900(27.35)	3709(27.32)	3491(23.95)	
**Education, n (%)**							<0.001
Grades 0–12	9284(16.12)	2072(18.79)	1909(16.82)	1864(16.69)	1718(14.52)	1721(14.12)	
High school graduate/GED	8650(24.30)	1751(26.03)	1768(24.89)	1721(23.64)	1713(23.32)	1697(23.83)	
Some college or above	19248(59.58)	3607(55.18)	3768(58.29)	3856(59.66)	3998(62.15)	4019(62.04)	
**Family Income to Poverty Ratio**^b^, **n (%)**							<0.001
<1.3	10914(20.74)	2539(25.42)	2241(21.03)	2137(20.21)	2065(19.46)	1932(18.13)	
1.3–3.5	14121(34.95)	2842(37.25)	2873(35.20)	2790(34.04)	2751(33.26)	2865(35.24)	
≥3.5	12147(44.32)	2049(37.33)	2331(43.77)	2514(45.74)	2613(47.27)	2640(46.64)	
**Smoking**^c^**, n (%)**							<0.001
Non-smokers	19845(52.88)	3473(44.66)	3865(51.11)	4157(54.48)	4234(56.98)	4116(56.18)	
Former smokers	9452(24.98)	1800(24.14)	1792(22.97)	1812(24.38)	1874(24.60)	2174(28.63)	
Current smokers	7885(22.14)	2157(31.20)	1788(25.93)	1472(21.14)	1321(18.42)	1147(15.19)	
**Alcohol intake, n (%)**^d^							<0.001
Non-alcohol use	11542(25.61)	2344(26.36)	2243(24.86)	2212(24.13)	2280(25.22)	2463(27.51)	
Mild alcohol use	12525(35.74)	2351(32.76)	2486(34.70)	2522(35.81)	2555(37.19)	2611(37.85)	
Moderate alcohol use	5662(17.20)	1124(17.20)	1116(17.21)	1181(17.64)	1128(17.32)	1113(16.65)	
Heavy alcohol use	7453(21.45)	1611(23.67)	1600(23.23)	1526(22.42)	1466(20.27)	1250(17.99)	
**BMI, kg/m**^**2**^^f^**, mean (SE)**	28.65(0.07)	29.32(0.12)	28.99(0.13)	28.61(0.13)	28.54(0.12)	27.88(0.11)	<0.001
**Weight status, n (%)**^f^							<0.001
BMI <30	23489(65.24)	4340(60.54)	4541(62.92)	4747(65.11)	4823(66.65)	5038(70.32)	
BMI ≥30	13693(34.76)	3090(39.46)	2904(37.08)	2694(34.89)	2606(33.35)	2399(29.68)	
**HEI-2015**^e^**, mean (SE)**	50.52(0.21)	49.65(0.29)	50.03(0.28)	50.69(0.29)	50.66(0.31)	51.44(0.29)	<0.001
**HEI-2015**^e^**, n (%)**							<0.001
<44.06	12394(34.02)	2583(36.60)	2545(35.99)	2429(33.42)	2451(33.17)	2386(31.28)	
44.07–56.18	12394(33.31)	2430(32.89)	2499(32.53)	2515(33.51)	2483(33.75)	2467(33.80)	
≥56.19	12394(32.67)	2417(30.51)	2401(31.48)	2497(33.07)	2495(33.08)	2584(34.93)	
**Platelet, mean (SE)**	256.52(0.79)	211.97(1.02)	241.86(1.17)	253.82(1.08)	272.00(1.02)	297.11(1.42)	<0.001
**Lymphocyte, mean (SE)**	2.15(0.01)	2.99(0.03)	2.42(0.01)	2.11(0.01)	1.88(0.01)	1.48(0.01)	<0.001
**PhenoAge, years, mean (SE)**	43.75(0.21)	43.47(0.35)	42.19(0.32)	42.70(0.37)	43.32(0.35)	47.00(0.36)	<0.001
**Diabetes (n,%)**	5594(10.47)	1421(14.32)	1192(10.99)	1045(10.14)	925(8.33)	1011(9.02)	<0.001
**Hypertension(n,%)**	15713(36.63)	3283(38.66)	3054(35.39)	3000(35.21)	2980(34.87)	3396(39.18)	<0.001
**Cancer**	3446(9.20)	633(8.98)	573(7.76)	616(8.63)	672(8.78)	952(11.76)	<0.001
**CVD**	3643(7.63)	840(9.55)	672(7.33)	637(6.46)	645(6.70)	849(8.30)	<0.001

Abbreviations: PLR, Platelet-lymphocyte ratio; GED, General Equivalency Diploma; HEI, Healthy Eating Index; BMI, Body mass index; SE, standard error; NHANES, National Health and Nutrition Examination Survey; PhenoAge, phenotypic age; CVD, cardiovascular.

a Means and percentages were adjusted for survey weights of NHANES.

b Family income to poverty ratio represents the ratio of family income to the poverty threshold, adjusted for household size.

c Cigarette smoking was defined as smoking at least 100 cigarettes during their lifetime, with former smokers defined as not currently smoking and current smokers defined as currently smoking.

d Alcohol use was defined as having at least 12 alcohol drinks in any given year. Heavy alcohol use (≥3 drinks per day for females, ≥4 drinks per day for males, or binge drinking [≥4 drinks on same occasion for females, ≥5 drinks on same occasion for males] on 5 or more days per month); Moderate alcohol use (≥2 drinks per day for females, ≥3 drinks per day for males, or binge drinking ≥2 days per month).

e HEI-2015 was calculated to measure adherence to the 2015 Dietary Guidelines for Americans with a higher score corresponding to a higher-quality diet.

f BMI was calculated by dividing weight in kilograms (kg) by height in meters squared (m^2^). Participants were classified normal weight (BMI <30 kg/m^2^), and obese (BMI ≥30 kg/m^2^).

### 3.2 The relationship between the PLR and mortality

The median follow-up time was 9.5 years [interquartile range (IQR): 5.3–14.1 years]. A total of 5683 deaths occurred [median (IQR) time to death: 7.0 (3.8–10.9) years], with 1792 deaths from cardiovascular diseases, 1289 from cancer, and 431 from respiratory disease, with a median time to death of 7.0 (3.8–11.0) years, 6.6 (3.4–10.3) years, and 7.3 (4.0–11.4) years, respectively.

### 3.3 Survival analysis

The association between the PLR and all-cause and cause-specific mortality is illustrated in Table 2. After controlling for all confounding variables in Model 3, the PLR in Quintile 5 was linked with greater risks of death from all-cause (*HR*, 1.14; *95 % CI*: 1.04–1.25, *P* for trend = 0.002), CVD (*HR*, 1.26; *95 % CI*: 1.01–1.57, *P* for trend = 0.015) and respiratory disease (*HR*, 1.98; *95 % CI*: 1.35–2.90, *P* for trend <0.001) than the PLR in Quintile 1. The risk of death from cancer was approximately 28 % lower for participants with PLRs in the fourth quintile than for participants with PLRs in the lowest quintile, respectively.

**Table 2:** HR (95 % CI) for all-cause and cause-specific mortality according to PLR

	PLR (HR, 95%CI)					P for trend
	Quintile 1 <89.62	Quintile 2 89.63–110.39	Quintile 3 110.40–131.25	Quintile 4 130.26–162.07	Quintile 5 ≥162.08	
**All-cause mortality**						
Death	1056	951	989	1070	1617	
Weighted death (%)	11.71	10.93	10.52	11.40	16.40	
Unadjusted Model 1	1[Reference]	0.82(0.70,0.96)	0.75(0.67,0.84)	0.76(0.66,0.88)	1.05(0.94,1.19)	0.018
Model 2	1[Reference]	0.95(0.83,1.08)	0.89(0.79,1.00)	0.91(0.80,1.03)	1.06(0.96,1.16)	0.056
Model 3	1[Reference]	0.99(0.87,1.13)	0.93(0.83,1.05)	0.96(0.84,1.10)	1.14(1.04,1.25)	0.002
**CVD mortality**						
Death	307	309	299	351	526	
Weighted death (%)	3.28	3.20	2.74	3.84	5.02	
Unadjusted Model 1	1[Reference]	0.85(0.68,1.07)	0.70(0.56,0.88)	0.91(0.70,1.20)	1.16(0.94,1.42)	0.013
Model 2	1[Reference]	1.01(0.81,1.24)	0.83(0.63,1.09)	1.10(0.85,1.42)	1.14(0.91,1.41)	0.078
Model 3	1[Reference]	1.08(0.88,1.32)	0.90(0.69,1.18)	1.19(0.91,1.56)	1.26(1.01,1.57)	0.015
**Cancer mortality**						
Death	255	229	251	220	334	
Weighted death (%)	2.97	2.86	2.84	2.29	3.33	
Unadjusted Model 1	1[Reference]	0.85(0.66,1.09)	0.81(0.65,1.00)	0.61(0.46,0.81)	0.85(0.70,1.04)	0.132
Model 2	1[Reference]	0.96(0.75,1.23)	0.95(0.75,1.19)	0.71(0.54,0.94)	0.87(0.70,1.07)	0.090
Model 3	1[Reference]	0.98(0.76,1.26)	0.95(0.76,1.21)	0.72(0.54,0.96)	0.87(0.71,1.07)	0.074
**Respiratory disease mortality**						
Death	84	60	59	81	147	
Weighted death (%)	0.82	0.80	0.71	1.03	1.78	
Unadjusted Model 1	1[Reference]	0.85(0.54,1.34)	0.72(0.46,1.13)	0.97(0.62,1.51)	1.61(1.09,2.35)	<0.001
Model 2	1[Reference]	1.04(0.64,1.71)	0.89(0.56,1.42)	1.23(0.79,1.93)	1.84(1.25,2.71)	<0.001
Model 3	1[Reference]	1.09(0.68,1.75)	0.94(0.61,1.47)	1.33(0.85,2.09)	1.98(1.35,2.90)	<0.001
**Other mortality**						
Death	410	353	380	418	610	
Weighted death (%)	4.65	4.06	4.22	4.24	6.27	
Unadjusted Model 1	1[Reference]	0.76(0.60,0.97)	0.76(0.61,0.95)	0.71(0.58,0.87)	1.01(0.83,1.23)	0.232
Model 2	1[Reference]	0.88(0.72,1.08)	0.89(0.72,1.09)	0.84(0.69,1.02)	1.00(0.84,1.20)	0.612
Model 3	1[Reference]	0.93(0.76,1.14)	0.93(0.76,1.15)	0.90(0.74,1.11)	1.10(0.92,1.31)	0.129

Model 2 was adjusted for age, sex, race/ethnicity, education level, family income to poverty ratio, smoking status, alcohol intake, Healthy Eating Index-2015 (<44.06, 44.06–56.18 or ≥ 56.19), and body mass index (<30 or ≥ 30).

Model 3 was additionally adjusted for history of hypertension, diabetes, cancer, and CVD.

Abbreviations: PLR, Platelet-lymphocyte ratio; HR, hazard ratio; CVD, cardiovascular.

### 3.4 The nonlinear association of restricted cubic splines between the PLR and mortality

**Fig. 2:**
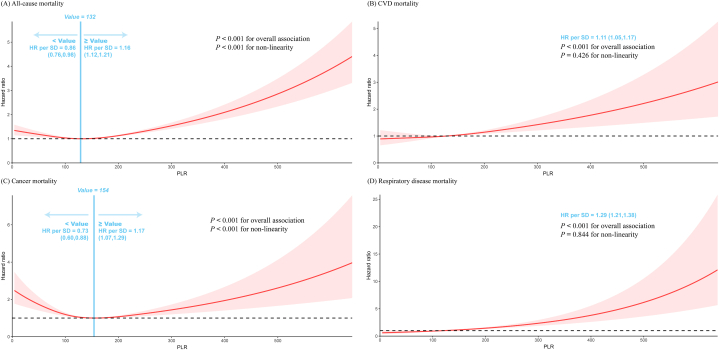
**Dose-response relationships between PLR and all-cause and cause-specific mortality.** Adjusted for age, sex, race/ethnicity, education level, family income to poverty ratio, smoking status, alcohol intake, Healthy Eating Index-2015, BMI, history of hypertension, diabetes, cancer, and CVD. The shaded part represents the 95 % CI. Abbreviations: PLR, Platelet-lymphocyte ratio; CVD, cardiovascular.

The association between the PLR and all-cause and cause-specific mortality was modeled and visualized with restricted cubic splines. The U-shaped associations were observed between the PLR and all-cause and cancer mortality (both *P* for overall <0.001, both *P* for non-linearity <0.001, Fig. 2 A and C). For every SD increase in the PLR, the all-cause mortality dropped by 14 % (HR, 0.86; 95 % CI: 0.76–0.98) if the PLR was <132, and increased by 16 % (HR, 1.16; 95 % CI: 1.12–1.21) if the PLR was ≥132. The cancer mortality decreased by 27 % (HR, 0.73; 95 % CI: 0.60–0.88) if the PLR was <154, but increased by 17 % (HR, 1.17; 95 % CI: 1.07–1.29) if the PLR was ≥154, for every SD increase in the PLR. Positively linear relationships were observed between PLR and CVD and respiratory disease mortality in whole participants (both *P* for overall <0.001, both *P* for non-linearity >0.05, Fig. 2 B and D). The mortality from CVD and respiratory disease increased by 11 % (*HR*, 1.11; *95 % CI*: 1.05–1.17) and 29 % (*HR*, 1.29; *95 % CI*: 1.21–1.38) for every SD increase in the PLR.

### 3.5 Associations of PLR with PhenoAge and PhenoAge with all-cause and cause-specific mortality

Table 3 displays the association of PLR with PhenoAge by the survey-weighted linear regression models. After adjustment for all covariates, the multivariate-adjusted βs of PhenoAge for every SD increase in PLR increased by 0.50 (95 % CI, 0.42–0.58), respectively. In addition, Table 3 also showed the association of PhenoAge with all-cause and cause-specific mortality by Cox regression models. The multivariate-adjusted HRs for every one-year increase in PhenoAge in the association with all-cause, CVD, cancer, and respiratory disease mortality were 1.05, 1.05, 1.05, and 1.06, respectively.

**Table 3:** Survey-weighted associations of PLR with PhenoAge and PhenoAge with all-cause and cause-specific mortality

Associations-	Univariable model		Model 1		Model 2	
	*Beta/HR* (*95%CI*)	*P* value	*Beta/HR* (*95%CI*)	*P* value	*Beta/HR* (*95%CI*)	*P* value
**PLR and PhenoAge, Beta (95%CI)**^a^	1.89(1.61,2.17)	<0.001	0.43(0.34, 0.52)	<0.001	0.50(0.42, 0.58)	<0.001
**PhenoAge and all-cause and cause-specific mortality, HR (95%CI)**^b^						
All-cause mortality	1.08(1.07,1.09)	<0.001	1.06(1.05,1.06)	<0.001	1.05(1.05,1.06)	<0.001
CVD mortality	1.09(1.08,1.10)	<0.001	1.06(1.05,1.07)	<0.001	1.05(1.04,1.06)	<0.001
Cancer mortality	1.07(1.06,1.07)	<0.001	1.04(1.03,1.05)	<0.001	1.05(1.04,1.06)	<0.001
Respiratory disease mortality	1.09(1.08,1.10)	<0.001	1.07(1.06,1.08)	<0.001	1.06(1.05,1.08)	<0.001

Model 2 was adjusted for age, sex, race/ethnicity, education level, family income to poverty ratio, smoking status, alcohol intake, Healthy Eating Index-2015 (<44.06, 44.06–56.18 or ≥ 56.19), and body mass index (<30 or ≥ 30).

Model 3 was additionally adjusted for history of hypertension, diabetes, cancer, and CVD.

Abbreviations: PLR, Platelet-lymphocyte ratio; PhenoAge, phenotypic age; HR, hazard ratio; CVD, cardiovascular.

a PLR as continuous variables (Per standard deviation increase).

b PhenoAge as continuous variables (Per 1 year increase).

### 3.6 Mediation analyses of PhenoAge on associations of PLR with all-cause and cause-specific mortality

**Fig. 3:**
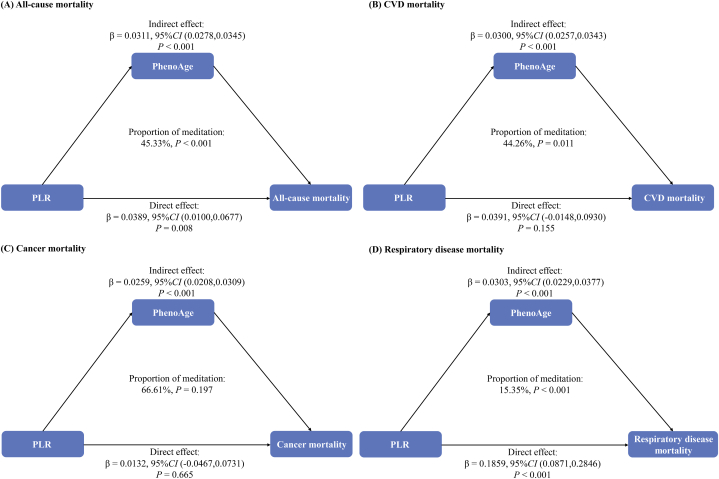
**The mediating proportion of PhenoAge on the associations between PLR and all-cause and cause-specific mortality.** Adjusted for age, sex, race/ethnicity, education level, family income to poverty ratio, smoking status, alcohol intake, Healthy Eating Index-2015, BMI, history of hypertension, diabetes, cancer, and CVD. Abbreviations: PhenoAge, phenotypic age; PLR, Platelet-lymphocyte ratio; CVD, cardiovascular.

Furthermore, causal mediation analyses were performed to evaluate the potential mediation effects of PhenoAge on the associations between PLR and all-cause and cause-specific mortality (Fig. 3; Table 4). PhenoAge had significant partial mediated effects on the associations of PLR with all-cause, and respiratory disease mortality, and the proportion of mediation was 45.33 %, and 15.35 %. PhenoAge had significant complete mediated effects on the associations of PLR with CVD, and cancer mortality, and the proportion of mediation was 44.26 %, and 66.61 %.

**Table 4:** The mediating proportion of PhenoAge on the associations between PLR^a^ and all-cause and cause-specific mortality

Model Pathways	Mediating Effect		
	*Beta Estimate* (95%*CI*)	*P* value	Proportion Mediated (%)
**All-cause mortality**			
Total effect	0.0700(0.0414,0.0985)	<0.001	100
Direct effect	0.0389(0.0100,0.0677)	0.008	54.67
Indirect effect	0.0311(0.0278,0.0345)	<0.001	45.33
**CVD mortality**			
Total effect	0.0691(0.0159,0.1224)	0.011	100
Direct effect	0.0391(-0.0148,0.0930)	0.155	55.74
Indirect effect	0.0300(0.0257,0.0343)	<0.001	44.26
**Cancer mortality**			
Total effect	0.0391(-0.0197,0.0978)	0.193	100
Direct effect	0.0132(-0.0467,0.0731)	0.665	33.39
Indirect effect	0.0259(0.0208,0.0309)	<0.001	66.61
**Respiratory disease mortality**			
Total effect	0.2162(0.1190,0.3134)	<0.001	100
Direct effect	0.1859(0.0871,0.2846)	<0.001	84.65
Indirect effect	0.0303(0.0229,0.0377)	<0.001	15.35

Adjusted for age, sex, race/ethnicity, education level, family income to poverty ratio, smoking status, alcohol intake, Healthy Eating Index-2015 (<44.06, 44.06–56.18 or ≥ 56.19), body mass index (<30 or ≥ 30), history of hypertension, diabetes, cancer, and CVD.

Abbreviations: PLR, Platelet-lymphocyte ratio; PhenoAge, phenotypic age; HR, hazard ratio; CVD, cardiovascular.

a PLR as continuous variables (Per standard deviation increase).

### 3.7 Sensitivity analyses

In three sensitivity studies, the results maintained robust after excluding deaths with a follow-up period of fewer than one year (Supplementary Table 3), excluding participants over 80 years old (Supplementary Table 4), and repeating the main analyses without consideration of complex sampling designs (Supplementary Table 5).

## 4 Discussion

An extensive, multiracial cross-sectional study of American adults revealed a relationship between the PLR level and overall and cause-specific death. According to the above findings, the PLR was independently associated with death from all causes, including CVD, cancer, and respiratory diseases. The above conclusions remained true even after the variables had been adjusted. We discovered the U-shaped associations between the PLR and mortality from all causes and cancer, and the positively linear links between the PLR and mortality from CVD and respiratory diseases. Furthermore, the study highlighted the potential of maintaining optimal PLR in mitigating PhenoAge and subsequently reducing mortality risk. Mediation analyses further supported the hypothesis that PhenoAge partially mediated the associations between PLR and all-cause, CVD, respiratory disease mortality. The results emphasize that primordial and primary prevention efforts to promote PLR should be strengthened to reduce the risk of early mortality in the later stages of life.

Prior research presented the relationship between PLR and all-cause mortality without further analysis [23]. However, we unexpectedly obtained a U-shaped curve association between the PLR and all-cause and cancer mortality. We found that the optimal PLR value with the lowest mortality risk was 132 in all-cause mortality. The PLR was negatively correlated with all-cause mortality when the PLR was <132 and positively correlated when the PLR was >132 (Fig. 2. A). Equally, we discovered that 154 for cancer mortality was the ideal PLR value with the lowest mortality risk (Fig. 2. C). To the best of our knowledge, there has been no related report that the PLR has a U-shaped relationship with all-cause and cancer mortality. However, many studies have revealed that platelet counts have clear U-shaped associations with all-cause and cause-specific mortality. For example, P.J. Vinholt et al. discovered a U-shaped correlation between mortality and platelet levels within 100–450 × 10^9^/L. Furthermore, both low and high platelet counts lead to an increase in total mortality [24]. In addition, the largest cohort examined for the relationship between abnormal platelet counts and mortality among older people was recruited by Ming-Tsun Tsai et al. They demonstrated that platelet counts exhibit U-shaped correlations with all-cause and cause-specific mortality without other risk factors [25]. The optimal PLR in our study suggested that maintaining PLR around a certain value might effectively decrease the risk of all-cause and cancer mortality. Slightly different, in contrast to the U-shaped relationships with all-cause and cancer mortality, we discovered the linear links between the PLR and mortality from CVD and respiratory diseases, consistent with earlier research. For example, Basem Azab et al. reported that a high PLR was related to poor CVD outcomes. Long-term (4-year) mortality following non-ST-elevation myocardial infarction was significantly and independently predicted by a high PLR (PLR >176) [26]. Zhuanbo Luo et al. reported that an elevated PLR indicated that patients with acute chronic obstructive pulmonary disease exacerbation would have a higher risk of 28-day death. These might be independent prognostic biomarkers [27].

Although the exact mechanism by which a high PLR may lead to high mortality is unclear, the PLR synthesizes thrombotic and inflammatory mechanisms [28]. Many experimental data have proven that active platelets increase thrombus formation in response to atherosclerotic plaque rupture or endothelial cell erosion, exacerbating atherothrombotic illness [29,30]. Inflammation inhibits platelet anti-adhesion properties, frequently leading to more interactions between platelets and the endothelium [31]. The release of proinflammatory mediators such as interleukin (IL)-1, IL-3, and IL-6 enhances megakaryocyte proliferation and results in thrombocytosis [32]. This situation causes a cascade of inflammatory reactions comparable to thrombosis and hemostasis [33].

Moreover, inflammation could boost lymphocyte apoptosis, leading to a high risk of infection and adverse cardiovascular disease consequences [34]. Nevertheless, why did our studies show that a low PLR predicts more significant mortality from cancer and all causes? We hypothesized that individuals with low platelet counts might also have poor regulatory capacity and lower lymphatic system inhibition, resulting in a low PLR and high mortality. Although intriguing, our conclusions and hypotheses need to be verified in other study populations. Based on the above mechanism, we believed that the PLR, as a biomarker of concurrent thrombosis and inflammation, could represent the degree of both conditions simultaneously and was a more accurate predictor of death than utilizing these two biomarkers separately.

Table 1 indicated that the PhenoAge of PLR varied significantly at different levels (*p* < 0.001). In Table 3, linear regression models and Cox regression models were used to verify the effect of PhenoAge on PLR and mortality. According to the mentioned findings, we further performed mediation analyses. The results showed that PhenoAge mediated the association between PLR and all-cause and cause-specific mortality. PhenoAge significantly mediated the associations of PLR and all-cause mortality, consistent with Zuyun Liu et al.’s research that PhenoAge was significantly associated with all-cause mortality after adjusting for chronological age and sex [15]. Additionally, they found in another study that certain behaviors had an impact on PhenoAge in persons with coronary artery disease, to which disadvantaged life course circumstances had the largest contribution. These findings strongly support the hypothesis that PhenoAge may ultimately have significant mediated effects on the associations of PLR with cardiovascular disease mortality [35]. In addition, we discovered comparable outcomes in respiratory disease. Márquez-Salinas A et al. believed that Adaptive metabolic and inflammatory responses identified using PhenoAge are linked to adverse outcomes in severe SARS-CoV-2 infection [36]. We have not yet determined the mediating role of phenoAge in the correlation between PLR and cancer mortality, considering cancer is more closely correlated with genetic and immune variables. These hypotheses and conclusions need to be verified in other study populations.

PLR is a well-known marker of systemic inflammation, where an elevated PLR reflects both increased platelet activity (pro-inflammatory) and decreased lymphocyte count (weakened immune function). Chronic inflammation is a driver of aging and is involved in the pathogenesis of several age-related diseases such as cardiovascular diseases, cancer, and autoimmune disorders [37,38]. Elevated PLR has been strongly associated with worse outcomes in aging populations, particularly due to its role in promoting thrombosis and atherosclerosis [39]. Inflammation plays a significant role in accelerating PhenoAge, as chronic inflammatory states (e.g., elevated PLR) can worsen cellular aging and lead to systemic deterioration. As such, PhenoAge captures the cumulative damage from prolonged inflammation [37]. This is key in linking PLR to mortality: as PLR rises, it accelerates biological aging (reflected in a higher PhenoAge), which in turn increases the risk of mortality.

Several regularly used inflammatory indices are available, including CRP levels, WBC counts, procalcitonin levels, and the erythrocyte sedimentation rate [40,41]. Despite the specificity and sensitivity of these indicators, monitoring them in clinical practice is not inexpensive. However, the PLR is relatively simple, and routine blood tests are inexpensive. It has evolved as a straightforward, affordable, easily measured, and accessible marker that could offer risk assessment for many inflammatory disorders.

There were four strengths in our research. To our knowledge, this is the first complete exploration of the mediated effects of PhenoAge on the associations of PLR with mortality. Second, this prospective cohort study used generalizable data from the NHANES, which enabled the research findings to be applied to a larger population. Moreover, we included 37,182 participants, leading to a large sample size. Third, we adjusted for various potential confounders, including BMI, lifestyle factors, demographic status, past medical history, and socioeconomic status. Fourth, our research spanned a more prolonged follow-up period (median, 9.5 years). Finally, we employed the R project and weighted Cox regression analysis rather than conventional Cox regression to obtain a reliable conclusion.

This study was also limited in several respects. First, the PLR was based on a one-time assessment lacking dynamic variable data. Second, although we considered several possible confounders and the cohort was a substantial sample, residual confounding could not be eliminated, especially from unmeasured factors. Third, the cross-sectional design of this study did not allow us to investigate the causal relationship between PLR and mortality due to its inherent limitations in capturing temporal changes and potential selection bias. Therefore, further rigorous Prospective Cohort Study, Experimental Study or Randomized Controlled Trials are required to establish a more definitive causal link and enhance the robustness of our findings.

## 5 Conclusion

The PLR was independently correlated with all-cause, cancer, cardiovascular, and respiratory mortality among US general adults and is an essential parameter that should be recommended for use. This study also discovered a novel U-shaped association between the PLR and all-cause and cancer mortality. Furthermore, this study revealed that PhenoAge partially mediates the relationships between PLR and all-cause and cause-specific mortality, providing valuable insights into the pathways linking PLR and mortality risk.

## CRediT authorship contribution statement

**Xiangjun Li:** Writing – original draft, Data curation. **Jia Wang:** Software, Resources, Formal analysis, Data curation. **Mengqi Zhang:** Writing – review & editing, Supervision, Project administration. **Yujing Li:** Writing – original draft, Supervision, Project administration. **Xiaoxuan Li:** Visualization, Resources, Investigation. **Jiaqi Zhang:** Validation, Software. **Lihua Zhang:** Writing – original draft, Resources, Methodology. **Yixuan Zhang:** Writing – review & editing, Resources, Investigation. **Zhenkang Qiu:** Writing – review & editing, Funding acquisition, Conceptualization.

## Ethics approval and consent to participate

The protocols of NHANES were approved by the institutional review board of the National Center for Health Statistics, CDC. Written informed consent was obtained from each participant before participation in this study.

## Consent for publication

Not applicable.

## Data availability statement

Data associated with this study has been deposited into a publicly available repository: the US National Health and Nutrition Examination Survey (NHANES), https://www.cdc.gov/nchs/nhanes/index.htm.

## Funding

Natural Science Foundation of Shandong Province (ZR2023QH190).

## Declaration of competing interest

The authors declare that they have no known competing financial interests or personal relationships that could have appeared to influence the work reported in this paper.

## Figures and Tables

**figs1:**