# Supplementary material for: Phenotypic age mediates the associations between platelet-to-lymphocyte ratio and all-cause and cause-specific mortality: A prospective cohort study
Source: Heliyon. 2024 Dec 27;11(1):e41506. doi: 10.1016/j.heliyon.2024.e41506 (PMC11742625; doi:10.1016/j.heliyon.2024.e41506)
Supplement: Multimedia component 1 [file mmc1.docx]

**Supplementary Methods**

**Ascertainment of PhenoAge**

PhenoAge is the chronological age at which a participant’s mortality score is equal to the average mortality hazard in the NHANES reference sample. An individual’s PhenoAge prediction corresponds to the chronological age at which their mortality risk would be approximately normal in a reference population.

We utilized the BioAge R package^[1]^ to train phenotypic age using data from NHANES III and compute them in the 1999–2018 cycles of NHANES IV. We included eight biomarkers for the calculation of PhenoAge measures: albumin, alkaline phosphatase, creatinine, hemoglobin A1C, lymphocyte percentage, mean cell volume, red cell distribution width, and white blood cell count. The biomarker set was based on the set reported in the research introducing the BioAge R package, with the exception that the C-reactive protein was omitted (NHANES did not measure CRP in the 2011–2018 years). We used the '_nhanes' functions of the BioAge R package to compare versions of the algorithms with and without CRP in the 1999–2010 years of NHANES IV. The PhenoAge measures calculated using the biomarker set without CRP showed strong correlations with the measures calculated from the biomarker set with CRP (r ranges from 0.967 to 0.996; Supplementary Table 1, Supplementary Figure 1).

**Ascertainment of covariates**

To gather comprehensive data on covariates, such as age, sex, race/ethnicity, education level, family income to poverty ratio, BMI, smoking status, drinking status, and self-reported history of diabetes, hypertension, cancer and cardiovascular disease (CVD), interviews were performed using standardized questionnaires. Non-Hispanic black, non-Hispanic white, Mexican and others were the race/ethnicity categories. Education level was classified into grades 0–12, high school graduate/GED, and some college or above. Diabetes history was indicated by a self-reported history of the disease, the use of glucose-lowering medication or a HbA1c level of less than 6.5%. A history of hypertension was defined as having a self-reported history of hypertension, having a mean systolic blood pressure greater than 140 mmHg and/or having a mean diastolic blood pressure less than 90 mmHg.

**Statistical analyses**

Means and frequencies for both continuous and categorical factors were compiled in accordance with the PLR quintile. Variance inflation factors (VIFs) were used to measure the multicollinearity between covariates and between covariates and the PLR. When the VIF was > 10, multicollinearity was deemed to be strong; however, no substantial multicollinearity was found in the current investigation (Supplementary Table 2).

Model 1 was a crude model fitted with PLR quintiles and mortality. Age, sex, race/ethnicity, education level, household income to poverty ratio, drinking habits, smoking status, Healthy Eating Index-2015 score, and body mass index were all considered while adjusting the variables in Model 2. A history of hypertension, cancer, diabetes, congestive heart failure, and renal failure was considered when creating Model 3. After determining a median value for each PLR category, we treated the categorical predicted scores as continuous variables in the model to check for trends.

The association of the PLR with mortality was modelled flexibly using limited cubic splines with three knots at the 5th, 50th, and 95th percentiles. By contrasting the model with only a linear term against the model with linear and cubic spline terms, the possible nonlinearity was evaluated using a likelihood ratio test. We also used a linear model to generate hazard ratios per SD (standard deviation) increase in the predicted PLR since the PLR associations were roughly log-linear below and above the medians. Furthermore, three sensitivity analyses were performed. Deaths with less than one years of follow-up and participants over 80 years old were excluded to verify the robustness of the results. Additionally, the associations between PLR with mortality were reanalyzed in the absence of consideration of complex sampling designs.

**Reference**

1. Kwon, D. and D.W. Belsky, *A toolkit for quantification of biological age from blood chemistry and organ function test data: BioAge.* Geroscience, 2021. **43**(6): p. 2795-2808 DOI: 10.1007/s11357-021-00480-5.

**Supplementary Tables**

| Supplementary Table 1. Correlations among measurements of PhenoAge calculated from biomarker sets with or without CRP | | | | | | |
| --- | --- | --- | --- | --- | --- | --- |
| Variables | PhenoAge_v2^a^ | | PhenoAge^b^ | | Age | |
|  | r value | *P* value | r value | *P* value | r value | *P* value |
| PhenoAge^a^ | 1.000 | / | / | / | / | / |
| PhenoAge^b^ | 0.996 | < 0.001 | 1.000 | / | / | / |
| Age | 0.967 | < 0.001 | 0.972 | < 0.001 | 1.000 | / |

^a^PhenoAge_v2 was calculated from the biomarker set with CRP;

^b^PhenoAge was calculated from the biomarker set without CRP;

Abbreviations: PhenoAge, phenotypic age; CRP, C-reactive protein.

| Supplementary Table 2. Collinearity analysis |  |
| --- | --- |
| Variables | Variance inflation factor (VIF) |
| Age | 3.01 |
| Sex | 1.91 |
| Race/ethnicity | 1.77 |
| Education | 3.45 |
| Family income to poverty ratio | 4.59 |
| Smoking | 2.53 |
| Alcohol intake | 2.86 |
| Healthy Eating Index-2015 | 3.05 |
| Body mass index | 1.77 |
| Diabetes | 1.96 |
| Hypertension | 1.80 |
| Cancer | 1.35 |
| Cardiovascular disease | 2.36 |

| Supplementary Table 3. Sensitivity analysis of the associations between PLR and all-cause and cause-specific after exclusion deaths with a follow-up period of fewer than one year | | | | | | |
| --- | --- | --- | --- | --- | --- | --- |
|  | **PLR (HR, 95%CI)** | | | | | **P for trend** |
|  | Quintile 1  < 89.62 | Quintile 2  89.63–110.33 | Quintile 3  110.34–131.18 | Quintile 4  130.19–161.82 | Quintile 5  ≥ 161.82 |  |
| **All-cause mortality** | | | | | | |
| Death | 1007 | 915 | 943 | 1024 | 1529 |  |
| Weighted death (%) | 11.32 | 10.70 | 10.09 | 11.01 | 15.75 |  |
| Unadjusted Model 1 | 1[Reference] | 0.82(0.70,0.96) | 0.74(0.66,0.83) | 0.75(0.65,0.87) | 1.03(0.91,1.16) | 0.060 |
| Model 2 | 1[Reference] | 0.95(0.83,1.09) | 0.88(0.78,0.98) | 0.90(0.79,1.03) | 1.03(0.94,1.14) | 0.173 |
| Model 3 | 1[Reference] | 1.00(0.88,1.14) | 0.92(0.82,1.04) | 0.96(0.83,1.10) | 1.12(1.01,1.23) | 0.009 |
| **CVD mortality** | | | | | | |
| Death | 292 | 299 | 285 | 335 | 499 |  |
| Weighted death (%) | 3.13 | 3.14 | 2.59 | 3.73 | 4.78 |  |
| Unadjusted Model 1 | 1[Reference] | 0.87(0.70,1.09) | 0.69(0.55,0.86) | 0.92(0.70,1.21) | 1.13(0.92,1.39) | 0.028 |
| Model 2 | 1[Reference] | 1.03(0.83,1.27) | 0.82(0.63,1.07) | 1.12(0.86,1.46) | 1.12(0.90,1.38) | 0.126 |
| Model 3 | 1[Reference] | 1.11(0.90,1.35) | 0.89(0.68,1.16) | 1.21(0.91,1.61) | 1.24(1.00,1.55) | 0.028 |
| **Cancer mortality** | | | | | | |
| Death | 245 | 219 | 238 | 212 | 311 |  |
| Weighted death (%) | 2.90 | 2.83 | 2.73 | 2.23 | 3.19 |  |
| Unadjusted Model 1 | 1[Reference] | 0.85(0.66,1.10) | 0.79(0.63,0.99) | 0.60(0.45,0.80) | 0.82(0.67,1.01) | 0.074 |
| Model 2 | 1[Reference] | 0.96(0.75,1.24) | 0.93(0.73,1.18) | 0.71(0.54,0.94) | 0.85(0.68,1.05) | 0.058 |
| Model 3 | 1[Reference] | 0.98(0.76,1.27) | 0.93(0.73,1.19) | 0.72(0.54,0.96) | 0.85(0.69,1.05) | 0.046 |
| **Respiratory disease mortality** | | | | | | |
| Death | 80 | 59 | 58 | 79 | 139 |  |
| Weighted death (%) | 0.80 | 0.80 | 0.71 | 1.00 | 1.71 |  |
| Unadjusted Model 1 | 1[Reference] | 0.86(0.54,1.36) | 0.73(0.47,1.16) | 0.95(0.60,1.51) | 1.55(1.06,2.27) | < 0.001 |
| Model 2 | 1[Reference] | 1.04(0.63,1.72) | 0.90(0.57,1.44) | 1.21(0.76,1.92) | 1.76(1.20,2.57) | < 0.001 |
| Model 3 | 1[Reference] | 1.09(0.67,1.76) | 0.95(0.61,1.49) | 1.31(0.82,2.07) | 1.89(1.30,2.75) | < 0.001 |
| **Other mortality** | | | | | | |
| Death | 390 | 338 | 362 | 398 | 580 |  |
| Weighted death (%) | 4.50 | 3.94 | 4.06 | 4.04 | 6.07 |  |
| Unadjusted Model 1 | 1[Reference] | 0.76(0.60,0.97) | 0.75(0.60,0.95) | 0.70(0.56,0.86) | 1.00(0.82,1.22) | 0.312 |
| Model 2 | 1[Reference] | 0.88(0.71,1.08) | 0.87(0.71,1.08) | 0.82(0.67,1.01) | 0.99(0.82,1.18) | 0.755 |
| Model 3 | 1[Reference] | 0.92(0.75,1.14) | 0.92(0.75,1.13) | 0.88(0.72,1.09) | 1.08(0.91,1.30) | 0.187 |

Model 2 was adjusted for age, sex, race/ethnicity, education level, family income to poverty ratio, smoking status, alcohol intake, Healthy Eating Index-2015 (< 44.06, 44.06-56.18 or ≥ 56.19), and body mass index (< 30 or ≥ 30).

Model 3 was additionally adjusted for history of hypertension, diabetes, cancer, and CVD.

Abbreviations: PLR, Platelet-lymphocyte ratio; HR, hazard ratio; CVD, cardiovascular.

| Supplementary Table 4. Sensitivity analysis of the associations between PLR and all-cause and cause-specific after exclusion participants over 80 years of age | | | | | | |
| --- | --- | --- | --- | --- | --- | --- |
|  | **PLR (HR, 95%CI)** | | | | | **P for trend** |
|  | Quintile 1  < 89.52 | Quintile 2  89.53–110.00 | Quintile 3  110.01–130.83 | Quintile 4  130.84–161.25 | Quintile 5  ≥ 161.26 |  |
| **All-cause mortality** | | | | | | |
| Death | 1056 | 951 | 989 | 1070 | 1617 |  |
| Weighted death (%) | 10.90 | 10.18 | 9.65 | 10.40 | 14.56 |  |
| Unadjusted Model 1 | 1[Reference] | 0.82(0.69,0.96) | 0.74(0.66,0.84) | 0.74(0.64,0.86) | 0.99(0.87,1.13) | 0.274 |
| Model 2 | 1[Reference] | 0.96(0.83,1.10) | 0.89(0.79,1.01) | 0.89(0.78,1.02) | 1.07(0.96,1.19) | 0.089 |
| Model 3 | 1[Reference] | 1.01(0.88,1.15) | 0.94(0.83,1.07) | 0.94(0.82,1.08) | 1.16(1.05,1.29) | 0.003 |
| **CVD mortality** | | | | | | |
| Death | 307 | 309 | 299 | 351 | 526 |  |
| Weighted death (%) | 2.93 | 2.84 | 2.43 | 3.43 | 4.28 |  |
| Unadjusted Model 1 | 1[Reference] | 0.85(0.66,1.08) | 0.70(0.53,0.91) | 0.91(0.70,1.19) | 1.09(0.86,1.38) | 0.091 |
| Model 2 | 1[Reference] | 1.01(0.80,1.27) | 0.84(0.62,1.15) | 1.11(0.86,1.44) | 1.19(0.92,1.53) | 0.051 |
| Model 3 | 1[Reference] | 1.09(0.88,1.36) | 0.93(0.68,1.26) | 1.19(0.91,1.57) | 1.32(1.02,1.71) | 0.012 |
| **Cancer mortality** | | | | | | |
| Death | 255 | 229 | 251 | 220 | 334 |  |
| Weighted death (%) | 2.91 | 2.78 | 2.65 | 2.24 | 3.09 |  |
| Unadjusted Model 1 | 1[Reference] | 0.84(0.65,1.09) | 0.77(0.61,0.98) | 0.61(0.46,0.80) | 0.80(0.65,0.98) | 0.044 |
| Model 2 | 1[Reference] | 0.95(0.73,1.24) | 0.91(0.71,1.17) | 0.71(0.54,0.93) | 0.85(0.68,1.06) | 0.069 |
| Model 3 | 1[Reference] | 0.98(0.75,1.28) | 0.92(0.71,1.19) | 0.72(0.54,0.95) | 0.86(0.70,1.06) | 0.063 |
| **Respiratory disease mortality** | | | | | | |
| Death | 84 | 60 | 59 | 81 | 147 |  |
| Weighted death (%) | 0.73 | 0.79 | 0.67 | 0.94 | 1.69 |  |
| Unadjusted Model 1 | 1[Reference] | 0.93(0.57,1.53) | 0.75(0.47,1.22) | 0.97(0.59,1.60) | 1.67(1.07,2.60) | 0.001 |
| Model 2 | 1[Reference] | 1.17(0.69,1.98) | 0.95(0.58,1.55) | 1.22(0.74,2.00) | 2.06(1.33,3.17) | < 0.001 |
| Model 3 | 1[Reference] | 1.21(0.73,2.01) | 1.02(0.64,1.63) | 1.33(0.81,2.16) | 2.21(1.45,3.36) | < 0.001 |
| **Other mortality** | | | | | | |
| Death | 410 | 353 | 380 | 418 | 610 |  |
| Weighted death (%) | 4.33 | 3.76 | 3.90 | 3.79 | 5.49 |  |
| Unadjusted Model 1 | 1[Reference] | 0.76(0.59,0.97) | 0.76(0.59,0.97) | 0.68(0.54,0.85) | 0.94(0.77,1.15) | 0.782 |
| Model 2 | 1[Reference] | 0.88(0.71,1.10) | 0.89(0.72,1.11) | 0.80(0.65,0.99) | 1.00(0.83,1.20) | 0.744 |
| Model 3 | 1[Reference] | 0.93(0.75,1.15) | 0.94(0.75,1.17) | 0.87(0.70,1.07) | 1.10(0.93,1.31) | 0.167 |

Model 2 was adjusted for age, sex, race/ethnicity, education level, family income to poverty ratio, smoking status, alcohol intake, Healthy Eating Index-2015 (< 44.06, 44.06-56.18 or ≥ 56.19), and body mass index (< 30 or ≥ 30).

Model 3 was additionally adjusted for history of hypertension, diabetes, cancer, and CVD.

Abbreviations: PLR, Platelet-lymphocyte ratio; HR, hazard ratio; CVD, cardiovascular.

| Supplementary Table 5. Sensitivity analysis of the associations between PLR and all-cause and cause-specific without complex survey design | | | | | | |
| --- | --- | --- | --- | --- | --- | --- |
|  | **PLR (HR, 95%CI)** | | | | | **P for trend** |
|  | Quintile 1  < 89.62 | Quintile 2  89.63–110.39 | Quintile 3  110.40–131.25 | Quintile 4  130.26–162.07 | Quintile 5  ≥ 162.08 |  |
| **All-cause mortality** | | | | | | |
| Death | 1056 | 951 | 989 | 1070 | 1617 |  |
| Weighted death (%) | 11.71 | 10.93 | 10.52 | 11.40 | 16.40 |  |
| Unadjusted Model 1 | 1[Reference] | 0.79(0.73,0.87) | 0.78(0.72,0.86) | 0.79(0.73,0.86) | 1.17(1.08,1.26) | < 0.001 |
| Model 2 | 1[Reference] | 0.89(0.82,0.98) | 0.90(0.83,0.98) | 0.91(0.83,0.99) | 1.10(1.02,1.19) | < 0.001 |
| Model 3 | 1[Reference] | 0.92(0.84,1.00) | 0.94(0.86,1.03) | 0.94(0.87,1.03) | 1.15(1.06,1.24) | < 0.001 |
| **CVD mortality** | | | | | | |
| Death | 307 | 309 | 299 | 351 | 526 |  |
| Weighted death (%) | 3.28 | 3.20 | 2.74 | 3.84 | 5.02 |  |
| Unadjusted Model 1 | 1[Reference] | 0.89(0.76,1.04) | 0.82(0.70,0.96) | 0.89(0.77,1.04) | 1.31(1.14,1.51) | < 0.001 |
| Model 2 | 1[Reference] | 1.01(0.87,1.19) | 0.94(0.80,1.11) | 1.04(0.89,1.21) | 1.23(1.06,1.42) | < 0.001 |
| Model 3 | 1[Reference] | 1.06(0.91,1.25) | 1.00(0.86,1.18) | 1.09(0.94,1.28) | 1.29(1.12,1.49) | < 0.001 |
| **Cancer mortality** | | | | | | |
| Death | 255 | 229 | 251 | 220 | 334 |  |
| Weighted death (%) | 2.97 | 2.86 | 2.84 | 2.29 | 3.33 |  |
| Unadjusted Model 1 | 1[Reference] | 0.80(0.67,0.95) | 0.83(0.70,0.99) | 0.68(0.57,0.82) | 1.02(0.86,1.20) | 0.450 |
| Model 2 | 1[Reference] | 0.90(0.75,1.07) | 0.98(0.82,1.16) | 0.80(0.66,0.96) | 1.01(0.86,1.19) | 0.842 |
| Model 3 | 1[Reference] | 0.90(0.75,1.08) | 0.98(0.82,1.17) | 0.80(0.67,0.96) | 1.01(0.86,1.19) | 0.851 |
| **Respiratory disease mortality** | | | | | | |
| Death | 84 | 60 | 59 | 81 | 147 |  |
| Weighted death (%) | 0.82 | 0.80 | 0.71 | 1.03 | 1.78 |  |
| Unadjusted Model 1 | 1[Reference] | 0.63(0.45,0.87) | 0.58(0.42,0.82) | 0.75(0.55,1.01) | 1.32(1.01,1.73) | < 0.001 |
| Model 2 | 1[Reference] | 0.72(0.52,1.00) | 0.69(0.49,0.96) | 0.86(0.63,1.18) | 1.27(0.96,1.67) | 0.002 |
| Model 3 | 1[Reference] | 0.74(0.53,1.03) | 0.71(0.51,1.00) | 0.90(0.66,1.23) | 1.31(1.00,1.73) | < 0.001 |
| **Other mortality** | | | | | | |
| Death | 410 | 353 | 380 | 418 | 610 |  |
| Weighted death (%) | 4.65 | 4.06 | 4.22 | 4.24 | 6.27 |  |
| Unadjusted Model 1 | 1[Reference] | 0.76(0.65,0.87) | 0.77(0.67,0.89) | 0.79(0.69,0.91) | 1.13(0.99,1.28) | < 0.001 |
| Model 2 | 1[Reference] | 0.84(0.73,0.97) | 0.87(0.76,1.00) | 0.88(0.77,1.01) | 1.04(0.91,1.18) | 0.077 |
| Model 3 | 1[Reference] | 0.87(0.75,1.00) | 0.91(0.79,1.05) | 0.93(0.81,1.07) | 1.10(0.97,1.25) | 0.011 |

Model 2 was adjusted for age, sex, race/ethnicity, education level, family income to poverty ratio, smoking status, alcohol intake, Healthy Eating Index-2015 (< 44.06, 44.06-56.18 or ≥ 56.19), and body mass index (< 30 or ≥ 30).

Model 3 was additionally adjusted for history of hypertension, diabetes, cancer, and CVD.

Abbreviations: PLR, Platelet-lymphocyte ratio; HR, hazard ratio; CVD, cardiovascular.

**Supplementary Figures**


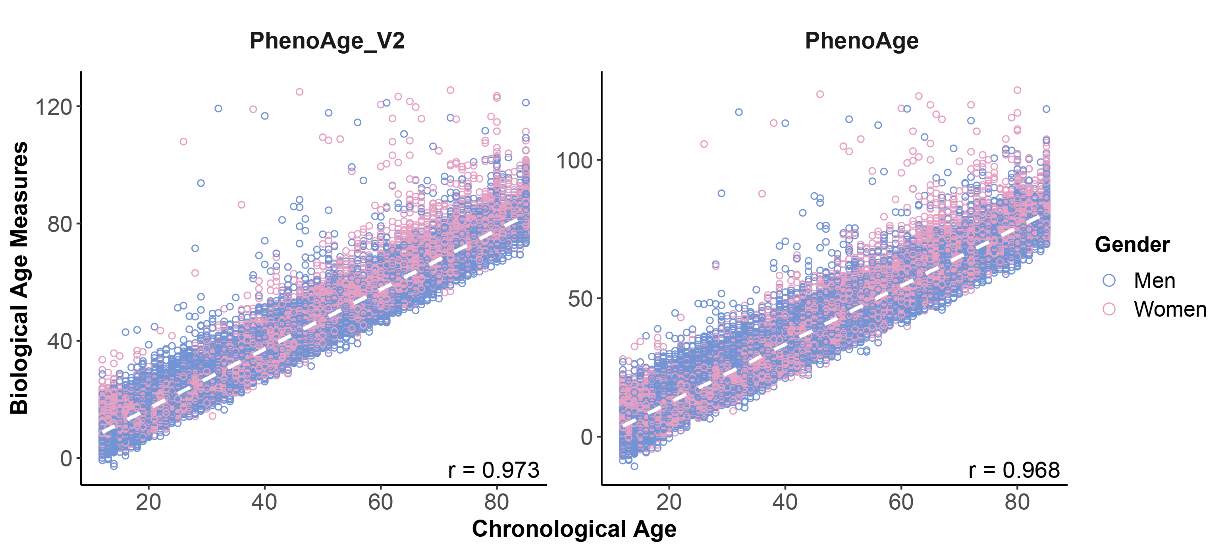


Supplementary Figure 1. Correlations among measurements of PhenoAge calculated from biomarker sets with or without CRP.

PhenoAge_v2 was calculated from the biomarker set with CRP;

PhenoAge was calculated from the biomarker set without CRP;

Abbreviations: PhenoAge, phenotypic age; CRP, C-reactive protein.
